# Ultra-Rapid Access to Words in Chronic Aphasia: The Effects of Intensive Language Action Therapy (ILAT)

**DOI:** 10.1007/s10548-014-0398-y

**Published:** 2014-11-18

**Authors:** Lucy J. MacGregor, Stephanie Difrancesco, Friedemann Pulvermüller, Yury Shtyrov, Bettina Mohr

**Affiliations:** 1 Medical Research Council, Cognition and Brain Sciences Unit, 15 Chaucer Road, Cambridge, CB2 7EF UK; 2 Department of Psychology, Anglia Ruskin University, Cambridge, UK; 3 Department of Philosophy and Humanities, Freie Universität Berlin, Berlin, Germany; 4 MINDLab – Center of Functionally Integrative Neuroscience, Institute for Clinical Medicine, Aarhus University, Aarhus, Denmark; 5 Faculty of Psychology, Higher School of Economics, Moscow, Russia; 6 Department of Psychiatry, Campus Benjamin Franklin, Charite University Medicine, Berlin, Germany

**Keywords:** Aphasia, Language therapy, MEG, ILAT/CIAT, Mismatch negativity, Stroke

## Abstract

Effects of intensive language action therapy (ILAT) on automatic language processing were assessed using Magnetoencephalography (MEG). Auditory magnetic mismatch negativity (MMNm) responses to words and pseudowords were recorded in twelve patients with chronic aphasia before and immediately after two weeks of ILAT. Following therapy, Patients showed significant clinical improvements of auditory comprehension as measured by the Token Test and in word retrieval and naming as measured by the Boston Naming Test. Neuromagnetic responses dissociated between meaningful words and meaningless word-like stimuli ultra-rapidly, approximately 50 ms after acoustic information first allowed for stimulus identification. Over treatment, there was a significant increase in the left-lateralisation of this early word-elicited activation, observed in perilesional fronto-temporal regions. No comparable change was seen for pseudowords. The results may reflect successful, therapy-induced, language restitution in the left hemisphere.

## Introduction

Language impairments, or aphasias, are amongst the most common and devastating cognitive problems resulting from strokes and other brain injuries (Pedersen et al. 1995). Although most stroke victims show improvements of language functions within the first few months of recovery, many patients are left with chronic post-stroke aphasia, which entails communication problems in daily life and greatly diminished quality of life. Functional recovery after stroke varies greatly between individuals. The degree and quality of cognitive changes can be attributed to different factors such as location and extent of brain lesions, motivation, and premorbid variables (Crosson et al. 2005; Lazar et al. 2008; Warburton et al. 1999).

 Following the initial recovery period, once aphasic patients have reached what is considered the chronic state (>1 year post stroke) they often do not have access to language therapy. However, research on brain reorganisation after stroke has demonstrated that even chronic aphasia patients can benefit from Constraint-Induced Aphasia Therapy (CIAT, see Pulvermüller et al. 2001a, Pulvermüller and Berthier 2008), a type of intensive language therapy more recently known as Intensive Language Action Therapy (ILAT; see Difrancesco et al. 2012). CIAT and ILAT refer to exactly the same therapy method; we now refer to CIAT as ILAT in order to highlight two of the main features of the therapy, namely that language practice is intensive (typically 3 h per day) and that language training takes place in action contexts where linguistic forms are used to perform communicative speech acts such as ‘making a request’ or ‘planning an action’ (for more details, see Difrancesco et al. 2012). The efficacy of ILAT/CIAT has been demonstrated and replicated in several randomised controlled clinical trials, showing that patients reliably improve a variety of different language functions such as naming, auditory comprehension, reading, and verbal repetition) after only two weeks of intensive therapy administered 3–4 h per day (Berthier et al. 2009, 2011; Meinzer et al. 2006; Pulvermüller et al. 2001a) and that these improvements are substantially above those achieved in conventional aphasia therapy (Pulvermüller et al. 2001b) and anomia treatment (Kurland et al. 2012 for evidence and discussion of the importance of the intensity of aphasia therapy on patient outcome, see Bhogal et al. 2003 and Cherney 2012). However, to date, few studies have investigated the effects of successful aphasia therapy on brain reorganisation, and, specifically, there has been little investigation of the specific cortical changes induced by ILAT in chronic stroke patients. Here, we explore the neuronal processes that underlie the reorganisation and restitution of linguistic and communicative function related to successful therapy in chronic post-stroke aphasia, by measuring the neurophysiological brain responses elicited in response to the processing of linguistic stimuli before and following ILAT.

The role of each hemisphere in supporting functional recovery of language after stroke, and related to successful therapy, is still somewhat unclear. In an fMRI study using an overt naming task, improvements in naming (for trained items only) following therapy (30 h over two weeks) was associated with increased functional activation of the perilesional regions of the left hemisphere that had been identified as dysfunctional (excessive slow wave activity) pre-therapy (Meinzer et al. 2008). Another overt naming fMRI study showed that successful language recovery after intensive intervention co-occurred with increased activation of both hemispheres (Kurland et al. 2012). In contrast, other studies using MEG (Breier et al. 2006) and fMRI (Richter et al. 2008) have shown that improvement in language function after therapy was correlated with greater relative activation in the right hemisphere before therapy. In an EEG study in which patients performed a lexical decision task before and after ILAT, therapy resulted in word-specific enhancements of the N250 response over both hemispheres (Pulvermüller et al. 2005), although the low spatial resolution of the EEG used in that study may not be sensitive enough for detecting topographic distinctions.

Data from several other studies suggest that contribution of the left and right hemispheres to language recovery may depend on the phase of recovery. In an fMRI study where participants detected semantic violations in short spoken sentences there was reduced activation of the unaffected left hemisphere areas in the acute phase following stroke, which was followed by an increase of activation and stronger involvement of right hemispheric areas at a later, more chronic state (Saur et al. 2006). Using MEG, whose spatial resolution is superior to EEG, one study (Breier et al. 2009) presented patients with spoken words in a recognition memory test, and reported early improvements in language function just one week following completion of ILAT (36 h over three weeks), accompanied by an increase in right posterior hemisphere activation 150–800 ms after word onset. However, three months after completion of the therapy, left hemispheric activations were observed in those patients who maintained improvements in function (Breier et al. 2009; see also Breier et al. 2007). Taken together, the evidence for functional recovery of language in aphasia in response to intensive language therapy suggests contributions of both hemispheres; it seems likely that differences in the localisation of therapy-related effects across different studies may be related to the variety of tasks and methods (see Berthier and Pulvermüller 2011).

When studying the effects of therapy-related brain reorganisation associated with language processing it is interesting to assess not only where in the brain these changes occur, but also their temporal aspects. Magnetoencephalography (MEG) or electroencephalography (EEG) are ideally suited to track the dynamic nature of speech processing because they enable measurement of the corresponding brain activity non-invasively with millisecond time resolution. Thus, MEG and EEG offer the potential to identify therapy-induced rapid transient changes in neural activity associated with language processing. Of these two methods, MEG provides not only the somewhat higher spatial resolution, but, crucially, is a much more patient-friendly technique with substantial advantages in terms of patient comfort during the recording as well as significantly reduced preparation time and efforts.

The earliest stages of lexical processing take place automatically, and their neurophysiological correlates can be measured even in the absence of focussed attention towards linguistic stimuli (Garagnani et al. 2009; MacGregor et al. 2012; Pulvermüller et al. 2001a). One of the most established methods to investigate automatic language processing with MEG or EEG is the passive oddball paradigm in which the mismatch negativity (MMN) is elicited. The MMN (or its magnetic counterpart, the MMNm) is an event-related brain response elicited in response to infrequent (deviant or oddball) acoustic stimuli randomly presented in a context of frequent (standard) stimuli (Näätänen and Alho, 1995), that occurs around 100–250 ms after stimulus onset with an activation focus over fronto-temporal areas (Näätänen et al. 2007). A large body of work has demonstrated that the MMN (difference between the standard and deviant stimuli) is enhanced for meaningful words compared to meaningless pseudowords (Endrass et al. 2004; Pettigrew et al. 2004; Pulvermüller et al. 2001b, 2004; Shtyrov and Pulvermüller 2002). This lexical MMN enhancement, which tends to occur around 100–200 ms after word recognition point is thought to index automatic neural activation of memory representations of known words that are not present for meaningless pseudo-words (see Shtyrov and Pulvermüller 2007a and Shtyrov 2010 for reviews). It has been argued that the robustness of these representations can explain their automatic activation even in a passive listening setting, where attention is not focused on the verbal input (Garagnani et al. 2008, 2009; Shtyrov et al. 2010). An important feature of the passive listening paradigm is that participants are not required to perform a task or even pay attention to the stimuli, which makes it particularly suited for studying the brain dynamics of lexical processing in neurological patients (Shtyrov et al. 2012), especially aphasics who may be particularly affected by fatigue and attention lapses or have difficulty comprehending and responding to demands imposed by active tasks such as reading.

Previous MMN research has demonstrated that, compared to controls, aphasic patients show reduced MMN responses to speech sounds (such as/ba/and/pa/) but largely intact responses to pure tone deviants (Aaltonen et al. 1993; Csépe et al. 2001; Ilvonen et al. 2004; Wertz et al. 1998). There is also evidence that patients show less left lateralisation of MMN responses than controls (Breier et al. 2007) or larger responses over the right than left hemisphere (Teki et al. 2013). To date, it is not clear how these abnormal responses might be altered through therapy. We set out to use MEG responses to matched words and pseudowords in a passive listening auditory oddball paradigm to investigate neural changes underlying language recovery in chronic aphasics undergoing intensive ILAT therapy.

In the present study, we investigated the spatiotemporal dynamics of rapid automatic lexical processing in aphasia patients, before and after two weeks of ILAT. Words and pseudowords were presented auditorily in a passive-listening MMNm oddball design, whilst patients viewed a silent film. In line with previous research on cortical reorganisation of ILAT, we expected to find improvements of language functions after ILAT accompanied by changes in word-specific cortical activation patterns. We were particularly interested in whether aphasia therapy might be associated with changes to brain responses previously associated with the earliest stages of lexical processing and in potentially different hemispheric contributions to these dynamics.

## Methods

### Participants

Twelve patients (3 females, mean age 57 years, range 26–76 years) with chronic non-fluent aphasia were tested immediately before and after participating in a two week intensive language-action therapy. All were recruited from self-help groups. Table 1 provides clinical and demographic data for each patient. Patients were selected according to the following criteria: (i) mild to moderate language impairment following a single stroke affecting the territory of the left middle cerebral artery (MCA), assessed by structural MRI scans, (ii) chronically aphasic patients (>1 year post-stroke) (iii) monolingual native speakers of English before stroke, (iv) right-handedness before stroke, as assessed with the Edinburgh Handedness Inventory (Oldfield 1971), (v) no sensory problems or significant cognitive deficits based on previous neurological examinations and medical report, and (v) no additional neurological diagnosis. Patients with severe comprehension deficits who were not able to fully engage in therapy were excluded. Importantly, only stable chronic patients were selected to exclude any effects due to spontaneous remission, and none of the patients received any additional speech and language therapy during the study. Hence, any functional changes and cortical reorganisation could be attributed to specific treatment effects. Only right-handed patients were selected in order to avoid any confounding factors with premorbid language lateralization. Since we were interested in laterality effects in cortical reorganisation, we wanted to select a group where left-hemisphere language dominance can be assumed for all patients. Language dominance is more variable in left-handers and therefore any laterality effects are more difficult to interpret (Szaflarski et al. 2002).

**Table 1:** Clinical and demographic data of 12 patients who participated in two weeks of ILAT and underwent MEG testing

Patient	Age at therapy (years)	Sex	Handedness	Duration of aphasia (months)	Aetiology
WT	65	Male	Right	80	Ischemia
NT	74	Male	Right	127	Ischemia
RP	40	Female	Right	19	Haemorrhage
BJ	69	Male	Right	25	Ischemia
SH	48	Male	Right	17	Ischemia
RK	72	Male	Right	32	Ischemia
FD	60	Female	Right	137	Ischemia
ES	26	Female	Right	165	Haemorrhage
JB	41	Male	Right	19	Ischemia
JB2	76	Male	Right	234	Haemorrhage
JW	54	Male	Right	20	Ischemia
RC	59	Male	Right	104	Ischemia
Mean (SD)	57 (15.6)			81.6 (72.1)	

All patients had previously suffered a cerebrovascular accident (CVA) in the region of the left middle cerebral artery

Across the patients, lesions involved a number of left-hemisphere regions around and extending from the Sylvian fissure, including the inferior and middle frontal gyrus, inferior and superior temporal gyrus, inferior parietal areas, hippocampus and left insula. The damage also extended to white matter, following the curve of the arcuate fasciculus.

Language was assessed with the Boston Diagnostic Aphasia Examination (BDAE, Goodglass and Kaplan 1972) and additionally with the Token Test (TT, De Renzi and Vignolo 1962). Scores from different subtests of the BDAE and the TT before and after the therapeutic intervention are presented in Table 2. The study was approved by the Cambridgeshire Local NHS Research Ethics (NRES) Committee.

**Table 2:** Average pre- and post-therapy scores (and standard errors) from the subcategories of the Boston Diagnostic Aphasia Examination (BDAE), and from the Token Test

	Pre-therapy	Post-therapy
Auditory Comprehension (BDAE)	83.67 (0.74)	85.17 (0.99)
Syntactic Processing (BDAE)	18.75 (1.27)	18.00 (1.43)
Boston Naming Test (BDAE)	28.58 (4.86)	33.00 (4.22)
Token Test	28.58 (4.77)	23.67 (3.45)

In the BDAE, higher scores indicate better performance: Auditory Comprehension (maximal score: 90), Syntactic Processing (maximal score: 32), Boston Naming Test (maximal score: 60). In the Token Test, lower scores (error scores) indicate better performance: Token Test (maximal error rate: 50)

### Intensive Language Action Therapy

Language therapy was administered for 3–4 h a day, on 10 consecutive week days. Sessions were delivered as a group setting with an average of 3 patients and 2 speech therapists per group. Language and communication skills were practised in language games where behaviourally relevant tasks (for example, making requests, planning an activity) were practised with the help of playing cards depicting objects and scenes. In line with our previous study, patients were required to restrict their communication to spoken language rather than relying on gesturing. For more details on the therapeutic procedures, see (Difrancesco et al. 2012).

## MEG Study

### Stimuli

There were two sets of acoustically-similar counterbalanced monosyllabic stimuli, which were successfully employed for investigating neurolexical processing previously (e.g., Garagnani et al. 2009). Each set comprised one standard token with a consonant–vowel (CV) structure (*bye, pie*) and two deviants, which were formed by the addition of a final unvoiced stop consonant/p/or/t/to the standard to create a real word (*bite, pipe*) or a pseudoword (*bipe, *pite). By varying the factor Lexicality (the status of a stimulus as word vs. as pseudoword) and acoustic–phonetic features of the stimuli independently in an orthogonal design, in which the same sounds (/p/,/t/) were presented in word and pseudoword contexts (Table 3), we were able to attribute differences in brain responses to our experimental variables of lexicality and session, ruling out any acoustic/phonetic stimulus confounds.

**Table 3:** Orthogonal variation of lexicality and acoustic–phonetic features across the two sets of stimuli

Condition (proportion of all stimuli)	Set 1	Set 2
Standard (83.4 %)	*bye*	*pie*
Deviant*/p/*(8.3 %)	*bipe	*pipe*
Deviant*/t/*(8.3 %)	*bite*	*pite

Each set comprised a standard and two deviants

Meticulous procedures were applied to guarantee that stimulus sets of words and pseudowords were matched exactly and orthogonalised for physical acoustic features and to ensure that they could first be recognised at exactly the same point in time. Multiple examples of the items were spoken in a randomised order by a female native British English speaker, and digitally recorded (sampling rate 44.1 kHz). To obtain the standard tokens, *bye* and *pie*, exemplars of the deviants were chosen and the syllable-final phonemes (/p/,/t/) extracted. The standards had the same fundamental frequency or F0 (272 Hz), and were adjusted to have equal duration (330 ms) and average sound energy or root-mean-square (RMS) power. Chosen exemplars of the critical syllable-final phonemes/p/and/t/had the same duration (80 ms) and were also normalised to match for average RMS power. These phonemes were cross-spliced onto each of the standards to create the four deviant stimuli, thus avoiding different co-articulation cues and minimising acoustic differences between stimuli. The silent closure time between the CV end of the standard and onset of the plosion of the final stop consonant in the deviants was adjusted to a value typical for English unvoiced (80 ms) stops.

This strictly controlled stimulus set allowed us to time-lock neural responses to the onset of the final stop consonant in the deviants (/t/or/p/) because this is the first point in time where the standards differ from the deviants and thus the earliest point in time at which the word or pseudoword deviant could possibly be recognised as being either a meaningful word or a meaningless syllable, and the earliest point in time when the standard and deviant stimuli diverge and thus the MMNm response per se can be triggered.

### Procedure

The study comprised two MEG sessions interspersed by a two week period of intensive language therapy. In each session, participants (n = 12) were seated within a dimly-lit magnetically shielded room (IMEDCO GMBH, Switzerland) and were asked to focus their attention on watching a silent nature film (Blue Planet or Planet Earth) without subtitles. The sounds were presented binaurally through plastic tubing attached to in-ear headphones using the MEG compatible sound-stimulation system (ER3A insert earphones, Etymotic Research, Inc., IL, USA). The sounds were presented binaurally through plastic tubing attached to in-ear headphones using the MEG compatible sound-stimulation system (ER3A insert earphones, Etymotic Research, Inc., IL, USA). The volume was set to a default level, which was judged to be comfortable by an individual with normal hearing. In addition, at the start of each experimental session a hearing threshold test was conducted to ensure the participant could hear sufficiently and approximately equally in both ears, and identify cases where headphones were not fitted correctly (for example). To this end, pure tones of 1,000 Hz (approximately in the middle of the hearing range) were played at various amplitudes to identify each participant’s individual hearing threshold, defined as the sound level at which the participant detected 50 % of the test tones. All participants detected at least 50 % of the sounds when they were attenuated by 50 dB relative to the default volume setting used in the experiment, indicating normal hearing.

The two stimulus sets were presented in separate blocks (order counterbalanced across participants). Each block contained 970 tokens: 810 tokens of the standard stimulus and 80 tokens of each deviant (16.6 % total deviant probability). Standard and deviant tokens were presented in randomised order, with the constraints that a deviant was always followed by at least one standard (which was not included in the analysis) and never by another deviant token, and each block started with 10 standards to establish the standard sequence (which were not included in the analysis). These constraints resulted in 640 standard tokens for the analysis. Stimuli were presented with a mean stimulus onset asynchrony (SOA) of 900 ms (jittered by ±20 in 10 ms steps) using E-Prime 2.0 software (Psychology Software Tools, Inc., Pittsburgh, PA, USA). In addition to taking part in the MMN study, patients also passively listened to a block of multiple words and pseudowords lasting approximately 8 min (data not reported here).

### MEG Recording

MEG data were recorded continuously (sampling rate 1,000 Hz, bandpass filter from 0.03 to 330 Hz) using a whole-head Vectorview system (Elekta Neuromag, Helsinki, Finland) containing 204 planar gradiometer and 102 magnetometer sensors. Head position relative to the sensor array was recorded continuously by using five Head-Position Indicator (HPI) coils that emitted sinusoidal currents (293–321 Hz). Vertical and horizontal electro-oculograms (EOGs) were monitored with electrodes placed above and below the left eye and either side of the eyes. Before the recording, the positions of the HPI coils relative to three anatomical fiducial points (nasion, left and right pre-auricular points) were digitally recorded using a 3-D digitiser (Fastrak Polhemus, Colchester, VA).

### MEG Data Processing and Analysis

To minimise the contribution of magnetic sources from outside the head and to reduce any within-sensor artifacts, the data from the 306 sensors were processed using the temporal extension of the signal-space separation technique (Taulu and Kajola 2005), implemented in MaxFilter 2.0.21 software (Elekta Neuromag): correlates of MEG signal originating from external sources were removed and compensation was made for within-block head movements (as measured by HPI coils).

Subsequent processing was performed using the MNE Suite (version 2.7.3, Martinos Center for Biomedical Imaging, Charlestown, MA, USA) and the Matlab 2009 programming environment (MathWorks, Natick, MA, USA). The continuous data for the four recording blocks (2 stimulus sets, 2 sessions) were epoched between −50 and 600 ms relative to the onset of the stimulus-final plosion for the deviants or corresponding silent period for the standards, baseline-corrected over the 50 ms period before the plosion, and bandpass-filtered between 0.1 and 70 Hz. Epochs were rejected when the magnetic field variation at any gradiometer or magnetometer exceeded 2,000 fT/cm or 3,500 fT respectively, or when voltage variation at either bipolar EOG electrode exceeded 150 µV. To correct for variation in head position across participants we calculated the average sensor array based on the epoched data files from all participants (four per participant corresponding to the four recording blocks), selected the individual data file closest to the average and then interpolated all data to this array. For each participant, average event-related magnetic fields were computed for each of the six stimuli and MMNms calculated by subtracting the associated standard from each of the four deviants. Separate averages were then calculated for the words and pseudowords in the pre- and post-therapy sessions. For statistical analysis, the event-related magnetic fields were quantified as the absolute amplitude of the 102 orthogonal planar gradiometer pairs by computing the square root of the sum of squares of the amplitudes of the two gradiometers in each pair:

$$MMN \,\,for\,\, each\,\, gradiometer \,\,pair = \sqrt {MMN\,\, grad \,\,1^{2} + MMN \,\,grad 2^{2} }$$

**Fig. 1:**
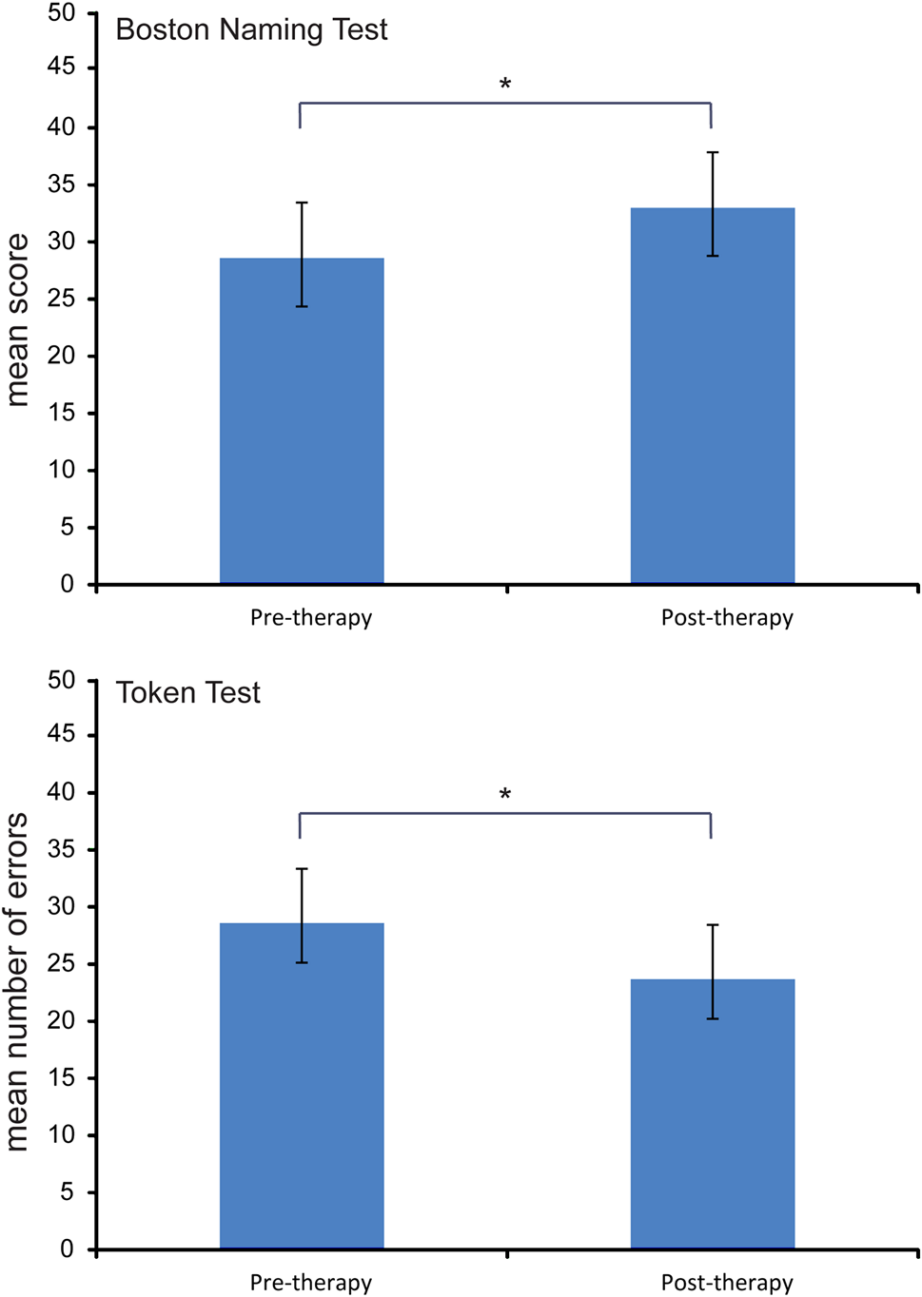
Bar charts showing significant improvements in performance in naming as measured by the Boston Naming Test (*top*) and in auditory comprehension as measured by the Token Test (*bottom*)

Such a calculation is essential to combine the data within each planar gradiometer pair because the gradients lie in orthogonal directions. Forty eight gradiometer channels (12 pairs over each hemisphere) positioned over fronto-temporal regions where responses were maximal were included in the analyses (see Fig. 1; Table 4). Amplitudes were statistically analysed using repeated measures analyses of variance (ANOVAs) with the factors Session (pre-therapy 1 vs. post-therapy) X Lexicality (words vs. pseudowords) X Hemisphere (left/LH vs. right/RH) X Location (Anterior vs. Central vs. Posterior) X Site (lateral vs. intermediate 1 vs. intermediate 2 vs. medial). The Greenhouse–Geisser correction for inequality of variance was applied where appropriate (data are reported with corrected *p* values). We chose three time windows for analysis, to capture the typical M50 (40–60 ms), the MMNm (100–150 ms) and the M300 (200–300 ms) responses.

**Table 4:** Forty eight sensors selected over fronto-temporal regions (12 gradiometer pairs in each hemisphere)

Site				
Location	Lateral	Inter 1	Inter 2	Medial
LH				
Anterior	0132/3	0213/2	0223/2	0413/2
Central	1513/2	0242/3	0233/2	0443/2
Posterior	1523/2	1612/3	1623/2	1813/2
RH				
Anterior	1122/3	1313/2	1323/2	1443/2
Central	1133/2	1343/2	1332/3	2613/2
Posterior	2223/2	2413/2	2422/3	2643/2

Numbers refer to the labelling of the Neuromag system

## Results

### Standardised Clinical Language Tests

Scores obtained from clinical language assessment obtained pre- and post-therapy were submitted to statistical analyses using paired t-tests. Patients showed significant improvements in language functions after the 10-day treatment interval in the Boston Naming Test (BNT), which is a subtest of the BDAE, t(11) = 3.08, *p* = 0.01 and in the Token Test (TT), t(11) = 2.53, *p* = 0.028 (Fig. 1). There was also a trend towards improvement in the Auditory Comprehension Test, which is a subtest of the BDAE, t(11) = 1.848, *p* = 0.09.

### MEG Activations

**Fig. 2:**
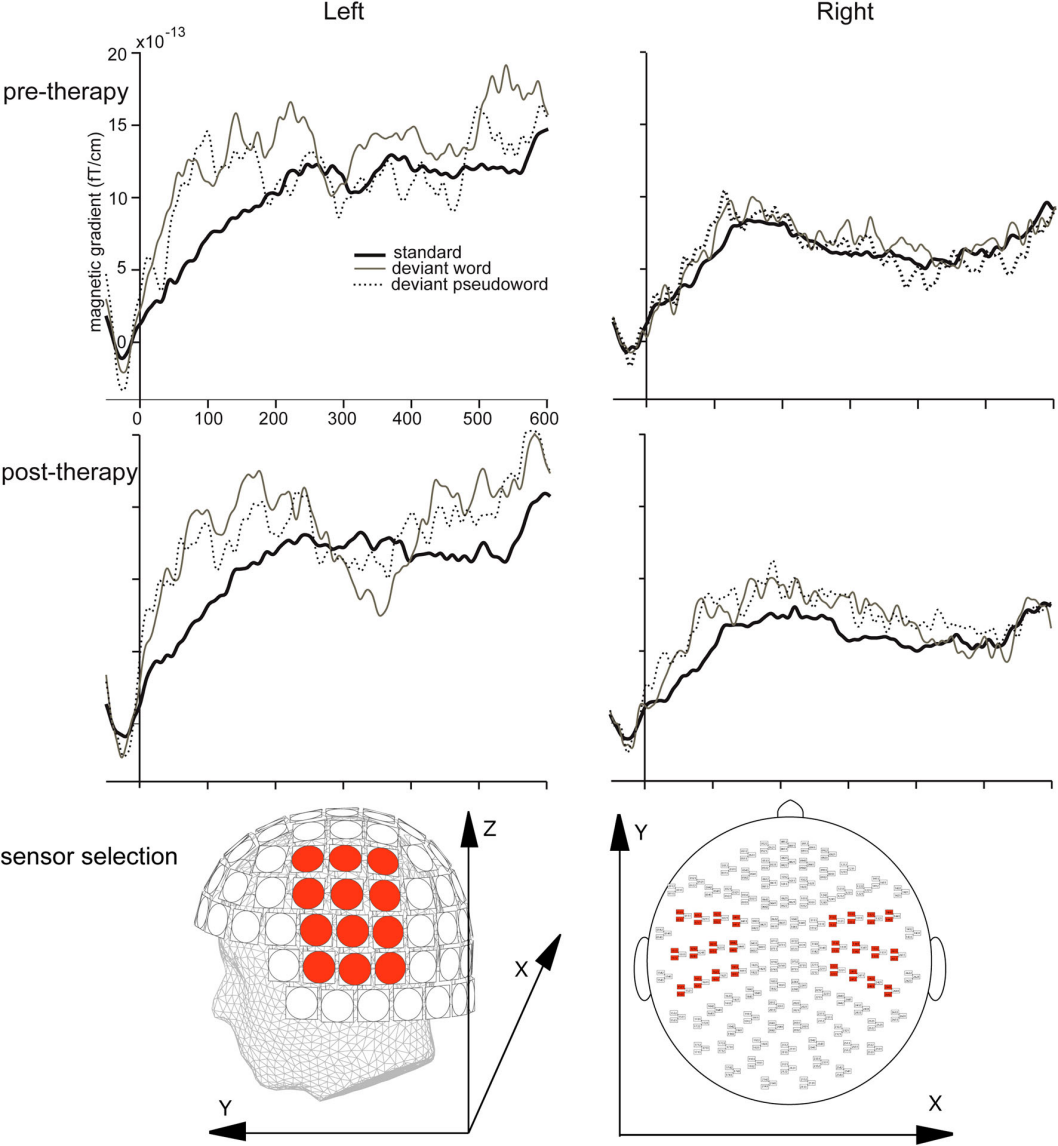
ERFs (n = 12) observed in response to standard stimuli (*black line*), deviant word stimuli (*grey line*) and deviant pseudoword stimuli (*dotted line*) before therapy (*top panel*) and after therapy (*middle panel*) for clusters of 12 gradiometer pairs in the left and right hemispheres (highlighted in the *lower panel*). For each gradiometer pair data were combined to give a single value by taking the square root of the sum of squares of the amplitudes of the two gradiometers. This calculation was performed separately for the averages of the standard and the deviant stimuli. Data are filtered between 0.1 and 70 Hz. *Lower panel* shows the location of the selected 24 gradiometer pairs over fronto-temporal areas of the *left* and *right* hemisphere

Figure 2 shows the event-related magnetic field gradients observed in response to standard stimuli, deviant word stimuli and deviant pseudoword stimuli before and after therapy. Both types of deviant stimuli elicited an increase in activation compared to the standard stimuli, which is particularly noticeable over the left hemisphere consistent with an MMNm response.

**Fig. 3:**
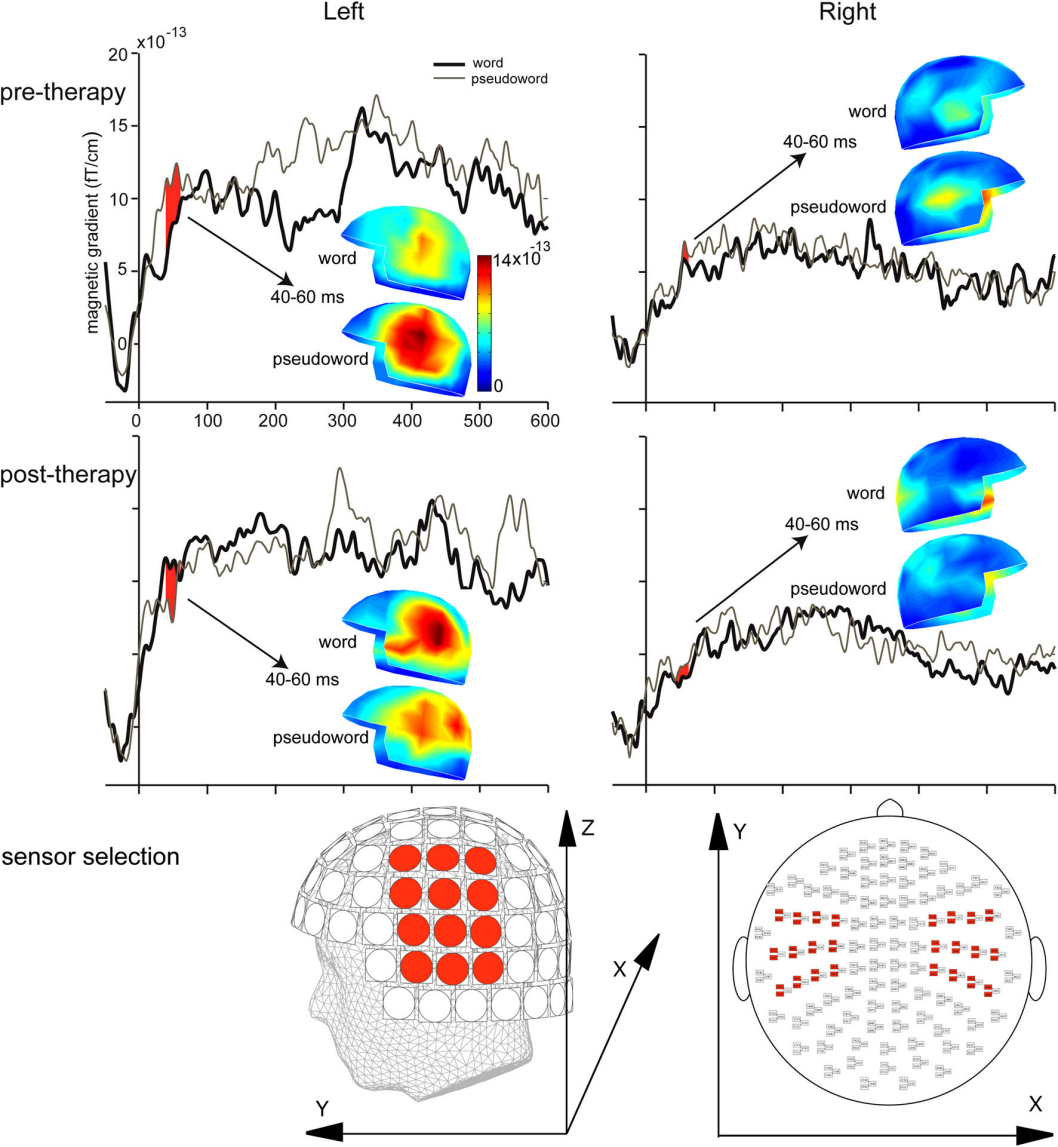
ERFs (n = 12) showing the MMNm responses (deviant minus standard) for words (*black line*) against pseudowords (*grey line*) before therapy (*top panel*) and after therapy (*middle panel*) for clusters of 12 gradiometer pairs in the left and right hemispheres (highlighted in the *lower panel*). For each gradiometer pair data were combined to give a single value by taking the square root of the sum of squares of the amplitudes of the two gradiometers. This calculation was performed separately for the averages of the MMNm responses for the words and the pseudowords. Data are filtered between 0.1 and 70 Hz. Topographical field gradient maps show the distribution of activations over the selected time window of 40–60 ms, for words and pseudowords, separately. *Lower panel* shows the location of the selected 24 gradiometer pairs over fronto-temporal areas of the *left* and *right* hemisphere

Analyses focused on the MMNm responses for words compared to pseudowords, which can be seen in Fig. 3. Note that the amplitude of the MMNm response differs to the amplitude difference between the standard and deviant responses shown in Fig. 2. This is because of the stage at which the non-linear RMS calculation, which was required to combine data within each planar gradiometer pair (see Methods), was performed. MMNm responses were compared between conditions (words/pseudowords and pre/post-therapy) in three time windows. Over a standard MMNm time window of 100–150 ms, an initial ANOVA (Session X Lexicality X Hemisphere X Location X Site) did not show any significant effects involving Lexicality. However, there was a main effect of Hemisphere, F(1,11) = 18.349, *MSE* = 353.398, *p* = 0.001, reflecting larger responses over the left (11.126 fT/cm) than right (6.381 fT/cm) hemispheres. There was also an interaction between Session and Location, F(2,22) = 4.018, *MSE* = 55.151, *p* = 0.036. Further exploration of this interaction did not reveal significant effects.

Over the M50 time window (40–60 ms) an initial ANOVA (Session X Lexicality X Hemisphere X Location X Site) revealed a significant three-way interaction between Session, Lexicality and Hemisphere, F(1,11) = 7.545, *MSE* = 82.820, *p* = 0.019, demonstrating significant differences between pre- and post- therapy effects for words and pseudowords across the hemispheres (see Fig. 3 and the accompanying topographic maps, which show the distribution of the responses). There were also marginally significant interactions between Session and Lexicality, F(1,11) = 3.744, *MSE* = 136.739, *p* = 0.079, as well as between Lexicality, Hemisphere and Location, F(2,22) = 3.189, *MSE* = 52.667, *p* = 0.070.

To explore the 3-way Session X Lexicality X Hemisphere interaction further, ANOVAs were calculated separately, for words and pseudowords. There were no significant effects for pseudowords. However, for words there was a significant interaction between Session and Hemisphere, F(1,11) = 5.858, *MSE* = 117.723, *p* = 0.034, indicating laterality differences in the processing of words, before and after treatment. There was also a main effect of Hemisphere, F(1,11) = 32.935, *MSE* = 136.362, *p* < 0.001. Analyses to follow up the two-way Session X Hemisphere interaction revealed a main effect of Hemisphere both before therapy, F(1,11) = 10.709, *MSE* = 77.547, *p* = 0.007: LH = 7.995 fT/cm; RH = 4.599 fT/cm, and after therapy, F(1,11) = 24.642, *MSE* = 176.539, *p* < 0.001: LH = 10.957 fT/cm; RH = 3.184 fT/cm, reflecting larger activation for words over the left than the right hemisphere (Fig. 3, topographical maps). To explore the laterality effects further, we calculated laterality quotients $LQ = \frac{{\left( {LH - RH} \right)}}{{\left( {LH + RH} \right)}}\; \times \;100$ for words both before and after therapy and subjected these to a non-parametric signed Wilcoxon signed rank tests. This analysis showed that left laterality significantly increased over therapy (median values: 22 vs. 56.5, Z = −2.903, *p* = 0.004).

The 3-way interaction was also explored by analysing the sessions separately. For the pre-therapy session only, there was a main effect of Lexicality, F(1,11) = 6.310, *MSE* = 91.201, *p* = 0.029, reflecting stronger activation for pseudowords (8.296 fT/cm) than words (6.297 fT/cm) and a main effect of Hemisphere, F(1,11) = 15.325, *MSE* = 231.010, *p* = 0.002, indicating higher activation over the left (9.775 fT/cm) compared to the right (4.817 fT/cm) hemisphere. When analysing activation in the post-therapy session only, a significant main effect of the factor Hemisphere was obtained, F(1,11) = 26.643, *MSE* = 220.634, *p* < 0.001, again reflecting higher activation over the left (9.932 fT/cm) compared with the right (3.542 fT/cm) hemisphere.

Over the final time window (200–300 ms), an ANOVA again revealed a main effect of Hemisphere, F(1,11) = 13.730, *MSE* = 13.730, *p* = 0.003, reflecting greater activation over the left (11.782 fT/cm) than the right (6.957 fT/cm) hemisphere and a significant interaction between Session and Location, F(2,22) = 4.169, *MSE* = 4.169, *p* = 0.042. Further exploration of the interaction did not result in significant effects.

### Correlations Between Clinical Language Tests and MEG Activation

Spearman rank correlations (two-tailed) were carried out between the clinical data (BDAE Auditory Comprehension Test, BDAE Boston Naming Test and Token Test) and the MEG activations for words and pseudowords in each of the three time windows. Correlations were performed on the pre-therapy/post-therapy data and the differences calculated between pre- and post-therapy (post-therapy minus pre-therapy) for the behavioural and neurophysiological data (right and left hemispheres). After correction for multiple comparisons, no correlations were significant.

## Discussion

The temporal dynamics of neuronal changes following intensive language action therapy were investigated in a group of stroke patients with chronic aphasia. ILAT was administered for ten days (two sets of five consecutive days) during a period of two weeks. Before and after intervention, patients underwent clinical language assessment and took part in a lexical mismatch negativity (MMNm) passive-listening task, whilst MEG was recorded. Analyses focused on the MMNm responses (deviant minus standard) for words and pseudowords. The results revealed most notably (i) significant improvements of naming and auditory comprehension after a short period of therapy, and (ii) ultra-rapid (50 ms post-deviation point) changes in cortical activation associated with the processing of words following therapy, manifest as an increase in left lateralisation of the MMNm word response over perilesional areas. Taken together, the findings demonstrate that ILAT improves aspects of language function and leads to changes in the automatic early stages of processing of words in fronto-temporal perilesional regions.

The present study replicates previous results in demonstrating improvement in linguistic skills even after only a short period of (intensive) language training (Berthier and Pulvermüller 2011; Pulvermüller and Berthier 2008). In line with previous studies (Pulvermüller et al. 2001a; Richter et al. 2008; Meinzer et al. 2008; Breier et al. 2009; Kurland et al. 2012), significant improvements in language functions in patients suffering from chronic post-stroke aphasia were observed. Significant clinical improvements were seen in patients’ ability to name object pictures as measured by the Boston Naming Test (BDAE subtest) and in patients’ auditory comprehension, as measured by the Token Test. ILAT focuses on practising communication and pragmatic aspects of language within a context in which patients are required to make requests and plan actions. This means that during the course of therapy key language functions including auditory comprehension, sentence planning, word retrieval and naming are not targeted in isolated tests but practised in situations more akin to those encountered in everyday life. The improvement in scores on the Boston Naming Test suggests that using these functions in the ILAT setting was sufficient to improve performance on a direct measure of naming that requires word retrieval. Improvement was also seen in scores on the Token Test, a general measure of receptive language abilities, in which patients are asked to follow instructions to manipulate different objects and are thus required to use auditory comprehension and syntactic processing skills. Although there was no significant improvement in performance on the Auditory Comprehension subtest of the BDAE, there was some indication of a trend.

Future research is required to investigate which factors are most relevant for achieving therapeutic success, but we suggest three features of ILAT that may be particularly important: (1) it targets patients’ use of communicative speech acts, focussing on embedding actions in the communicative context, rather than training linguistic utterances outside communicative context, (2) it uses constraints to focus patients on practising communicative skills relevant in daily life and (3) it applies an unusually intensive training schedule of 3 h per day for 10 days over two consecutive weeks.

MEG results demonstrated that after therapy there was an increase in the left-lateralisation of early cortical activation in response to words. Given the timing of the effect and the paradigm (passive listening) through which it was elicited, we suggest the effect may reflect recovery of automatic lexical processing in the left hemisphere. Previous research using a similar passive listening MMN paradigm with healthy adults has repeatedly shown left lateralised MMN responses to words, which were significantly larger in amplitude than those to pseudowords and thought to reflect automatic lexical processing (Pulvermüller and Shtyrov 2009; Shtyrov and Pulvermüller 2007b). The effect in the present study, which was apparent very early on, around 50 ms after there was sufficient acoustic information to distinguish between meaningful words and nonsense pseudowords, is earlier than left-lateralised lexicality effects observed previously in MMN paradigms and thus may be a distinct effect. We note the timing is very similar to an effect reported in a MEG study in which multiple words and pseudowords were presented in a non-MMNm passive listening paradigm while participants were asked to ignore the incoming stimuli and focus on a movie (MacGregor et al. 2012). In this study, a lexical dissociation between neural responses to words and pseudowords was reported at around 50 ms after the onset of acoustic information required for stimulus identification, which was argued to reflect automatic lexical processing. We chose to use the MMN paradigm because it is now well-established as a method for investigating the nature of lexical processing and linguistic representations, particularly for revealing the earliest automatic stages of spoken language processing (Pulvermüller and Shtyrov 2006)*. The particular benefits of the MMN paradigm are that it allows for strict control of acoustic and phonological parameters of the stimuli, minimal stimulus variance, precise time-locking of brain responses to linguistic events, and fully matched standard-deviant acoustic contrasts between conditions. Future work is clearly needed to replicate these early lexical effects across different paradigms and different population groups. For example, given the timing of the critical effect observed in the present study, which is earlier than the standard MMN response, it will be interesting to consider patient responses elicited in response to multiple words and pseudowords presented in a non-MMNm passive listening design.

Somewhat unexpected was the observation of larger responses for pseudowords than words observed before therapy in the M50 time window. We do not have a straightforward explanation for this finding, however we wish to emphasise that the main result is that the M50 responses to the words became more left lateralised following therapy, whereas there was no difference in the responses to the pseudowords before and after therapy. We suggest that the increase in left lateralisation of the responses to words may reflect some normalisation of language function. Although we did not see specific evidence of reliance on the right hemisphere before therapy (in the M50 and MMN time windows responses to words were left lateralised both before and after therapy), previous research suggested that chronically aphasic patients seem to rely more on the right hemispheric for processing speech (Saur et al. 2006; Teki et al. 2013). (Teki et al. 2013) showed that MMN responses in the right hemisphere were larger than those in the left and no different in amplitude to those in the left hemisphere of the controls. The reliance on the right hemisphere was further demonstrated using Dynamic Causal Modelling (DCM), which revealed stronger modulation of connections from the right primary auditory cortex to the right superior temporal gyrus and also between hemispheres from the left primary auditory cortex to right primary auditory cortex (Teki et al. 2013).

Left-lateralised increases in activation over temporal regions following ILAT have been previously observed in a spoken word recognition MEG study (Breier et al. 2009). However, in that study, effects were measured relative to the onset of the words (rather than word recognition point as in the present study): the earliest neurophysiological effect occurred approximately 150 ms after stimulus onset. Although the effect was left lateralised when patients were tested at three months following therapy, immediately following the completion of therapy the effect was right-lateralised. As discussed in the introduction, previous research presents a mixed picture with respect to the role of each hemisphere in language recovery following therapy. Although an association between language improvement and increases in activation in the left (perilesional) region has been reported (Meinzer et al. 2008), other studies have found bilateral increases in activation (Kurland et al. 2012; Pulvermüller et al. 2005). Differences in the lateralisation of therapy-related effects on word processing across different studies may, at least in part, be attributable to the task employed, general task demands and also to the category of the words tested (Berthier and Pulvermüller 2011). For example, strong evidence has been accumulated that different linguistic processes (lexical vs. syntactic) lead to different degrees of laterality and even the semantic properties of single spoken or written words can be manifest in the topographically specific left- or right-lateralised, or bilaterally symmetric brain processes (Mohr et al. 1994, 2007; Pulvermüller et al. 2009; Pulvermüller 2013). Further understanding of the brain processes and involvement of each hemisphere in language recovery would be gained from source level analysis, which we did not perform here due to lack of structural scans for most of the patients. Previous research has identified possible sources of the M50 bilaterally, in the auditory cortices (Huotilainen et al. 1998, Korzyukov et al. 2007, Ligeois-Chauvel et al. 1994), which is also where the auditory mMMN signal is thought to be generated (Alho 1995; Näätänen 2001). Evidence also suggests generators of the mMMN in the prefrontal cortex, with a left hemisphere bias for linguistic stimuli (Näätänen et al. 1997; Pulvermüller et al. 2001a).

In the standard MMNm time window we observed strongly left lateralised responses for both words and pseudowords, which contrasts with results from previous research using speech sounds (rather than whole words) showing no left lateralisation (Breier et al. 2007) or even right lateralisation (Teki et al. 2013) of MMN responses in aphasic patients. Given the large body of research with healthy adults demonstrating larger responses for words compared to pseudowords in the MMNm time window (for reviews, see Shtyrov and Pulvermüller 2007a; Shtyrov 2010) we had anticipated similar lexicality effects, which might be enhanced by the therapy. In fact these effects were absent in patients both before and after therapy, although visual inspection of the data after therapy suggests a tendency towards larger responses to words than pseudowords.

Some previous studies have reported correlations between MMN responses and clinical language functions (Ilvonen et al. 2003; Wertz et al. 1998) but after correction for multiple comparisons we did not find significant effects in any of the time windows. The lack of significance is perhaps not surprising because, as has been argued previously, the behavioural tests require active processing of language whereas mMMN responses reflect automatic processes (Breier et al. 2007). Recent research using DCM has, however, demonstrated a relationship between connectivity parameters measured in response to deviant speech sounds and clinical language functions. In this study, there was a negative correlation between inter-hemispheric connections (left superior temporal gyrus to right superior temporal gyrus) and behavioural performance on tests of phoneme discrimination, and a positive correlation between a feedback connection (right superior temporal gyrus to right primary auditory cortex) and a written sentence comprehension test (Teki et al. 2013).

One possible criticism of the design of the present study is the use of the same stimuli and tests in the behavioural and neurophysiological measures conducted before and after therapy, which means that the effects observed might, at least in part, relate to task and stimulus repetition effects. We believe, however, that this is unlikely for several reasons. The clinical language tests we used are established measures commonly used to to map progress over therapy. For example, the Token Test has been demonstrated to have good test–retest reliability (e.g., Huber et al. 1983). Neurophysiological repetition effects are typically manifest in reduced event-related potentials and fields (Young and Rugg 1992) whereas we observed an increase of early neuromagnetic activity over therapy. Furthermore, previous reports have shown stability in brain activation patterns across test–retest sessions in healthy controls within a 2-week interval (Meinzer et al. 2006), and in patients with aphasia who underwent a baseline scan three weeks before the pre-therapy scan (Breier et al. 2009). We therefore attribute the measured behavioural and neurophysiological effects to language therapy delivered between test and measurements.

In conclusion, the present study showed that intensive language action therapy (ILAT) is effective in treating patients with chronic aphasia, leading to significant improvements in word retrieval, naming and auditory comprehension abilities as measured by clinical language tests. Furthermore, following the short intensive therapy programme there was an increase in the left-lateralisation of fronto-temporal ultra-rapid neurophysiological brain responses to passively heard words compared to pseudowords, indicating cortical changes in the automatic processing of meaningful lexical stimuli.
